# On the relevance of cocaine detection in a fingerprint

**DOI:** 10.1038/s41598-020-58856-0

**Published:** 2020-02-06

**Authors:** M. Jang, C. Costa, J. Bunch, B. Gibson, M. Ismail, V. Palitsin, R. Webb, M. Hudson, M. J. Bailey

**Affiliations:** 1 Department of Chemistry, University of Surrey, Guildford, GU2 7XH UK; 2 Ion Beam Centre, University of Surrey, Guildford, GU2 7XH UK; 3 National Physical Laboratory, Teddington, TW11 0LW UK; 4 Forensic Science Ireland, Dublin, Republic of Ireland; 5 Intelligent Fingerprinting Limited, Milton Road, Impington, Cambridge, CB24 9NG UK

**Keywords:** Bioanalytical chemistry, Mass spectrometry, Medical and clinical diagnostics

## Abstract

The finding that drugs and metabolites can be detected from fingerprints is of potential relevance to forensic science and as well as toxicology and clinical testing. However, discriminating between dermal contact and ingestion of drugs has never been verified experimentally. The inability to interpret the result of finding a drug or metabolite in a fingerprint has prevented widespread adoption of fingerprints in drug testing and limits the probative value of detecting drugs in fingermarks. A commonly held belief is that the detection of *metabolites* of drugs of abuse in fingerprints can be used to confirm a drug has been ingested. However, we show here that cocaine and its primary metabolite, benzoylecgonine, can be detected in fingerprints of non-drug users after contact with cocaine. Additionally, cocaine was found to persist above environmental levels for up to 48 hours after contact. Therefore the detection of cocaine and benzoylecgonine (BZE) in fingermarks can be forensically significant, but do not demonstrate that a person has ingested the substance. In contrast, the data here shows that a drug test from a fingerprint (where hands can be washed prior to donating a sample) CAN distinguish between contact and ingestion of cocaine. If hands were washed prior to giving a fingerprint, BZE was detected only after the administration of cocaine. Therefore BZE can be used to distinguish cocaine contact from cocaine ingestion, provided donors wash their hands prior to sampling. A test based on the detection of BZE in at least one of two donated fingerprint samples has accuracy 95%, sensitivity 90% and specificity of 100% (n = 86).

## Introduction

Fingerprint samples are of interest in clinical testing, toxicology and forensic science. In forensics, it has been known for a long time that fingerprints carry more information than only their ridge details^1–3^, and interest in determining “activity level information” from a fingermark left at a crime scene has given impetus to numerous studies exploring drug detection from a fingerprint after contact with a substance^4–8^. There have also been a number of studies exploring the possibility of using a fingerprint to determine whether a donor has ingested a particular substance, i.e. for toxicology or clinical testing^9–14^ or in forensics, to show from a fingermark that a person has consumed or touched a drug^15^. In clinical testing, a fingerprint offers a promising new sampling matrix because of the ease and convenience of donating a sample^16^. Additionally, the ridge details that are embedded in the sample can be used to identify falsification and assure traceability.

In each of these cases, it is highly desirable to be able to distinguish dermal (touch) contact from ingestion of a substance, because the corresponding legal ramifications are different. Indeed, one reason fingerprint testing has not yet reached widespread acceptance from the clinical toxicology community is the possibility that a drug signal can arise from sources other than ingestion^17^. Whilst this concept has been comprehensively assessed for hair^18^ and oral fluid^19,20^ testing, very little data on the relevance of drug detection in fingerprints exists.

In recent work, Ismail *et al*. showed that cocaine could be detected in the fingerprints of drug users, and that it was possible to set a cut-off level to distinguish environmental exposure from cocaine use^21^. Our group has also researched rapid methods (~2 minutes per sample) for testing for cocaine from a fingerprint^22,23^, including paper spray mass spectrometry^24^, which we showed was compatible with visualisation of a fingerprint prior to analysis. This enables a sample to be collected, checked for authenticity and analysed within a timeframe of a few minutes.

Whilst previous work has explored the possibility of using a fingerprint to distinguish between cocaine ingestion and environmental exposure, there is currently no data on whether recent contact with cocaine can be distinguished from cocaine use. This lack of empirical data has precluded exploitation of the finding that a drug or metabolite can be detected in a fingerprint, because there is no interpretative framework. This means that the robustness of fingerprint samples for clinical drug testing is unproven, and also for forensic fingerprint evidence, it is not clear what finding a drug in a fingerprint actually means.

In this work, we explore for the first time the levels of cocaine and its primary metabolite, benzoylecgonine (BZE) observed in a fingerprint at various time intervals after contact with cocaine powder and with street cocaine. We compare these with fingerprint samples taken from a cohort of patients attending a drug rehabilitation clinic, as well as non-drug users. We show that it is possible to distinguish between contact and ingestion of cocaine from a fingerprint, if (and only if) fingerprints are donated after washing hands.

## Results

### Method validation

**Figure 1:**
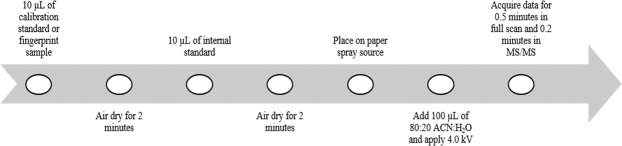
Paper spray mass spectrometry analysis workflow for the analysis of calibration standards and fingerprint samples.

A full validation of the paper spray high resolution mass spectrometry method developed for this work is provided in the Supporting Material and a schematic of the method is given in Fig. 1. For cocaine and BZE, the precision was <25%, R^2^ > 0.9847 for standards between 50 and 1,200 pg, LOD 15 pg (cocaine) and 50 pg (BZE). No instability of cocaine or BZE, or matrix effects caused by the presence of a fingerprint were observed.

### Fingerprint samples donated from non-drug users

**Figure 2:**
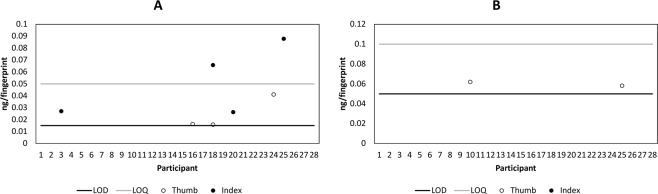
Mass per fingerprint of (**A**) cocaine and (**B**) benzoylecgonine detected in the fingerprints (right thumb and right index) from 28 non-drug users, collected as presented (AP) and measured using paper spray mass spectrometry.

Figure 2 shows the mass per fingerprint of BZE and cocaine detected in the fingerprints of non-drug users using paper spray mass spectrometry. Prior to handwashing, cocaine and BZE were detected in 7 and 2 fingerprint samples respectively, with no fingerprints containing both analytes. After handwashing, neither analyte was detected in any fingerprint sample.

### Fingerprint samples donated directly following contact with street cocaine

**Figure 3:**
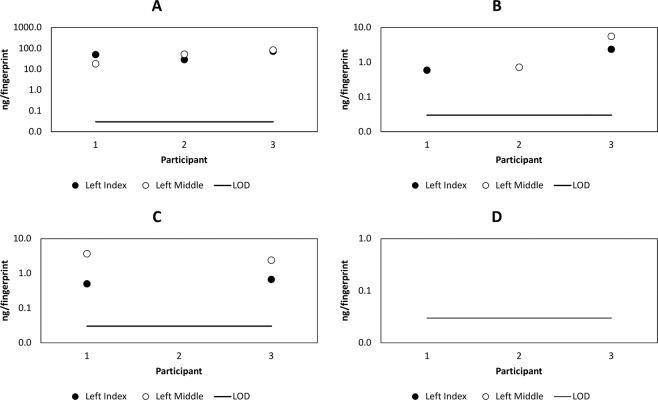
Mass of per fingerprint of cocaine (**A,C**) and benzoylecgonine (**B,D**) detected in fingerprints (left index and left middle) from 3 donors after contacting 2 mg street cocaine immediately after contact (**A,B**) and after washing hands (**C,D**).

Figure 3(A,B) shows the mass per fingerprint of BZE and cocaine detected in the fingerprints of 3 participants who touched street cocaine, directly after contact with the substance. An important observation is the detection of the metabolite, BZE in the fingerprint samples from donors who did not ingest cocaine. It has been hypothesised by ourselves and others^4,15,22^ that the metabolites of a drug can be used to confirm that a drug has been metabolised and excreted. These results show that the detection of a drug metabolite in a fingerprint provided from unwashed hands does not *necessarily* arise from drug ingestion. However, Fig. 3(C,D) shows that after hand washing, whilst cocaine is still observed in the fingerprint samples provided by Donors 1 and 3, BZE is no longer detected.

### Persistence of cocaine on the fingertips

**Figure 4:**
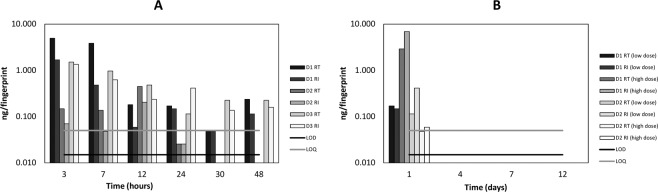
Mass per fingerprint of cocaine detected in fingerprints collected as presented (AP) from 3 donors (D1, D2 and D3 respectively), (**A**) at various time points up to 48 hours (short term) after touching 2 mg cocaine of 99% purity and (**B**) at various time points up to 12 days (long term) after touching 0.5 mg and 2 mg cocaine of 99% purity.

To obtain a clearer picture of the significance of detecting cocaine in a fingerprint, the longer term persistence of cocaine after contact with cocaine hydrochloride powder (99% purity) was studied. Figure 4 shows the mass of cocaine per fingerprint observed after various time periods up to 48 hours (short term persistence, Fig. 4A) and 12 days (long term persistence, Fig. 4B). Qualitative analysis of the cocaine powder dissolved in acetonitrile (to 700 ng/mL) showed a signal for BZE, but this was not detected in any fingerprint sample, confirming the previous result that BZE can be washed off hands, whilst cocaine persists. As shown in Fig. 4, cocaine was not observed to persist for longer than 48 hours.

**Figure 5:**
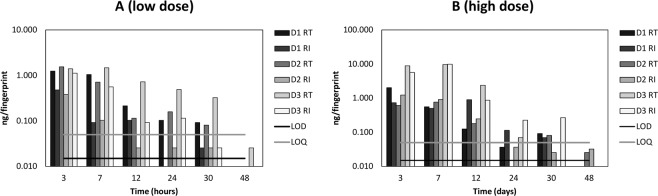
Mass per fingerprint of cocaine detected in fingerprints collected after washing hands (AH), of 3 donors (D1, D2 and D3 respectively) at various time points after touching (**A**) 0.5 mg and (**B**) 2 mg cocaine of 99% purity.

Figure 5 shows the short term (up to 48 hours) persistence of cocaine in a fingerprint after contact with a low (0.5 mg) and high (2 mg) dose of cocaine. In this instance, donors were asked to wash their hands prior to providing a sample. Neither increased cocaine dose nor handwashing prior to donation of a sample had a significant impact on the time window after which cocaine was observed in the fingerprints.

### Patient fingerprint samples

**Figure 6:**
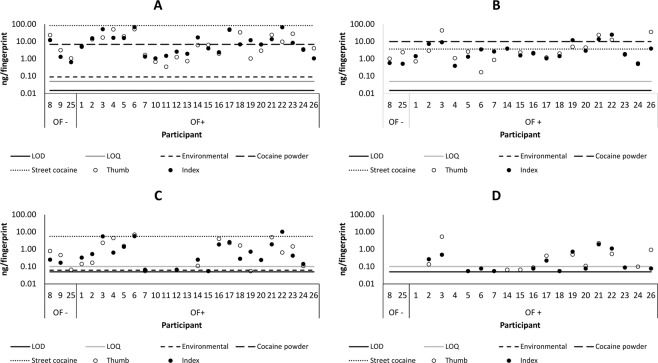
Mass per fingerprint of cocaine (**A,B**) and benzoylecgonine (**C,D**) detected in the fingerprints (right thumb and index) of drug users before (**A,C**) and after (**B,D**) handwashing. The samples are grouped according to the oral fluid test result, where OF- denotes a negative oral fluid test result, and OF + denotes a positive test result for cocaine. The lines represent the maximum mass of analyte observed in a fingerprint taken from the washed hands of (a) a non-drug user (from Fig. 2 -“environmental”); (b) after contact with cocaine hydrochloride powder (from Fig. 5B – “cocaine powder”) and after contact with street cocaine (Fig. 3C,D – “street cocaine”).

Figure 6 displays the mass of cocaine and BZE detected in patient fingerprints before and after handwashing^21^. The maximum mass of analyte observed in a fingerprint after contact with cocaine hydrochloride powder (from Figs. 4 and 5)/street cocaine (from Fig. 3) and is denoted by the line “cocaine powder”. Similarly, the maximum mass of analyte observed in a fingerprint from a non-drug user (from Fig. 2) is denoted by the line “environmental”. Consistent with the work of Ismail *et al*. using LC-MS^21^, cocaine is detected at elevated levels in the patient samples compared with the “environmental” level. The fact that the “street cocaine” line is higher than the “cocaine powder” line is explained by the fact that these samples were taken immediately after contact with street cocaine, whereas for cocaine powder, samples were taken 3 hours after contact. For either situation, no clear distinction between ingestion and contact can be made on the basis of cocaine detection alone, even after the patient has washed their hands.

A particularly interesting result comes from inspection of the fingerprint samples derived from Patients 8 and 25, whose oral fluid tested negative for cocaine. Before handwashing, both patients presented with cocaine and BZE in their fingerprints. After handwashing, whilst cocaine was still detected, BZE was no longer detected in either patient fingerprint. This, combined with the observation that contact residues of BZE are not observed following handwashing, suggests that BZE could be a more suitable target for fingerprint based drug screening than cocaine.

## Discussion

The data presented here show that the detection of cocaine in a fingerprint is indicative of either ingestion of cocaine OR recent contact with cocaine. The persistence study shows that >48 hours after contact, cocaine is no longer detected in a fingerprint by this method. The background study shows that provided hands are washed prior to giving a sample, cocaine is not normally detected in the fingerprints of non-drug users. Therefore whilst the detection of cocaine in a fingerprint does not necessarily indicate it has been ingested, it does show either ingestion or recent (within approximately 48 hours) contact with the drug. Similarly, the detection of BZE from unwashed hands shows either that a person has either consumed OR recently handled cocaine. Therefore detection of BZE or cocaine in a fingermark can be forensically significant, but does not show that a person has consumed cocaine.

It should be noted that for health and safety reasons, the dose of cocaine that could be touched was limited to 2 mg. Although no difference was observed in the length of time it took to remove detectable traces of 0.5 mg and 2 mg cocaine following contact, a limitation of this study is the inability to test higher doses to explore how long large doses of cocaine can persist on the fingers for.

In contrast, for drug testing, it does appear to be possible to distinguish between cocaine ingestion and contact. Provided hands were washed prior to giving a sample, BZE was ONLY observed in fingerprints of drug users whose oral fluid tested positive for cocaine. Even immediately after contact with street cocaine, BZE was not observed after hands were washed. Similarly, BZE was not detected amongst the non-drug users, or the drug users who tested negative in their oral fluid (provided hands were washed prior to giving a sample). For the patients whose oral fluid was positive for BZE, only 2 patients out of 19 did not return any fingerprints positive for BZE, giving a detection rate of 89.5%. A test based on the requirement for at least 1 fingerprint (of 2) to be positive for BZE therefore gives values of accuracy, sensitivity and specificity of 95%, 90% and 100% respectively (n = 86). Future work could explore whether a more sensitive method could be developed to improve on the sensitivity.

A further limitation of the study is the fact that the sample volume of a fingerprint is unknown, (also the case in previous work). Although attempts were made to control the deposition parameters (by controlling the pressure, time and area of deposition), the amount of sweat produced by a donor could not be controlled, and was not accounted for in any way. This undoubtedly leads to some variability, for example the intra donor variability that can be seen in Fig. 6. This currently limits the ability to provide a quantitative test result based on a fingerprint, and will be explored in future work. However, the data presented here shows that in spite of this, it is possible to distinguish between touch and ingestion of cocaine in a drug testing scenario.

## Conclusion

The results presented here have implications for both drug testing and the forensic analysis of fingerprints. It has been shown that detection of BZE or cocaine in a fingerprint collected from unwashed hands can mean either recent contact (<48 hours) or ingestion of cocaine. Therefore the detection of cocaine and BZE in a fingermark left at a crime scene means that either the substance was recently handled OR the person ingested the substance.

However, in a drug test, hands can be washed prior to donation of a sample, and here the detection of BZE was found to be solely due to the administration of cocaine. We conclude that cocaine can be used as a screening tool, because it is not readily detected at environmental levels, and was detected 100% of the time in samples from drug users. However, cocaine alone cannot be used to confirm drug ingestion, and BZE is a more appropriate candidate compound for distinguishing cocaine contact from cocaine ingestion, having 100% specificity, based on the samples tested here.

## Methods

All methods were carried out in accordance with relevant guidelines and regulations. All experimental protocols were approved by the National Research Ethics Service (NRES - REC reference: 14/LO/0346). Informed consent was obtained from all subjects.

### Chemicals and reagents

Drug standards (cocaine, benzoylecgonine (BZE)) and internal standards (cocaine-D3, and BZE-D3) were obtained from Cerilliant (Dorset, UK). To prepare all solution and solvent mixtures, LC-MS Optima^TM^ grade solvents: methanol (MeOH), acetonitrile (ACN), and water (H_2_O) were used in this work (Fischer Scientific, Leicestershire, UK). 0.1% (v/v) formic acid (Fischer Scientific, Leicestershire, UK) was added to all spray solvents (80:20/ACN:H_2_O) to enhance protonation. Cocaine hydrochloride powder (Sigma-Aldrich, UK), 99% purity was used for contact residue experiments.

Fingerprint samples were collected onto a paper substrate Whatman Grade 1 chromatography paper, cut into a triangle shape (1.6 × 2.1 cm, base × height), which had been washed with 0.1% hydrochloric acid and 50:50 (% v/v) MeOH:H_2_O and air dried. A glass slide was placed underneath the sample, and a folded aluminium foil was placed over the base of the triangle to minimise any carryover effects, as per our previous work (20).

### Instrumentation

Paper spray mass spectrometry analysis was carried out using a custom-made paper spray source built at the Surrey Ion Beam Centre. Paper spray mass spectrometry fundamentals are described elsewhere^25,26^. For analysis of fingerprints contaminated with street cocaine, the paper spray source was coupled to a Waters Micromass II Mass Spectrometer with paper spray analysis performed as described in Costa *et al*.^24^.

For all other experiments, the paper spray source was coupled to a Thermo Scientific™ Q.

Exactive™ Plus Hybrid Quadrupole-Orbitrap™ mass spectrometer (Thermo Scientific, UK). The spray voltage (4 kV), capillary temperature (250 °C), and S-lens RF level (50.0) were optimised using electrospray ionisation (ESI), based on the parameters that produced the highest protonated ion counts of analyte: cocaine (*m/z* 304.1539) and benzoylecgonine (*m/z* 290.1380). The paper spray parameters used are described in Fig. 1 and full details on the method validation is provided in the Supporting Material. The internal standard was 100 ng/ml cocaine-d3 and BZE-d3. The data acquisition was carried out for 0.5 minutes in full scan mode (*m/z* 66.7–1,000) for quantitative analysis, followed by 0.2 minutes in MS/MS mode to confirm peak assignment using the transitions *m/z* 304.1539 > 182.1178 and 82.0658 for cocaine and *m/z* 290.138 > 168.1020 for BZE with a normalised collision energy 35 (dimensionless).

The mass spectrometry parameters used in this study are listed in Supporting Material Data Table 1. All spectra were analysed using Xcalibur^TM^ (Thermo Fisher Scientific)^27^.

### Sample collection

In order to determine the most relevant hand preparation strategy, fingerprints were collected following two different scenarios: “as presented” (AP) and “after handwashing” (AH). For the “as presented” samples, participants deposited their fingerprints without any wiping or hand washing. For the “after handwashing” samples, participants washed their hands with soap and water and dried them completely using paper towels. They then wore gloves for 10 minutes to induce sweating. Samples were collected on the paper substrates using kitchen scales, pressing down until a reading of 1000 to 1200 g was reached for 10 seconds. Fingerprint samples were taken from 28 non-drug users (from the right thumb and right index finger) from the University of Surrey for both scenarios.

To investigate drug residues immediately after contact with cocaine, samples of cocaine seized by Irish police and stored at Forensic Science Ireland (FSI) were used. A sample from the drug seizure had been previously analysed by FSI’s standard analysis protocol using gas chromatography – mass spectrometry and had been found to have a cocaine purity of 41%, with traces of benzoylecgonine, ecgonine methyl ester, caffeine, lignocaine and levamisole (see Supporting Material). Three donors (non-drug users) were asked to touch 2 mg of the powder, spreading the powder over all their right hand fingers for approximately 10 seconds. They then dusted the excess off to remove large particles, and deposited fingerprints (left index and middle fingers) before and after handwashing, as described above.

To explore the short term persistence of cocaine using fingerprints, 3 donors (non-drug users) touched 2 mg cocaine hydrochloride powder (Sigma-Aldrich, UK), as described above. Participants deposited their fingerprints (right thumb and index) as described above after different time points: T = 3, 7, 12, 24, 30, and 48 hours following contact with cocaine hydrochloride powder. During each time period, participants carried out their usual daily activities, but there was no control on the number or timing of hand washes. To explore longer term persistence, the process above was repeated for 2 participants giving their fingerprints at 1, 4, 7 and 12 days after handling (a) 0.5 mg and (b) 2 mg cocaine hydrochloride respectively.

To explore further the possibility of eliminating cocaine signals from fingerprints following contact with the substance, the short-term persistence study was repeated, with participants being asked to provide fingerprint samples after washing their hands and wearing gloves (as described above).

To observe the range of levels of cocaine and BZE in the fingerprints of drug users, fingerprint samples (right thumb and right index) were collected from 26 patients attending a drug rehabilitation clinic, who testified administration of cocaine within last 24 hours. Fingerprints were provided as presented (from all 26 donors) and after handwashing (from the 21 donors who participated), as described above for collecting fingerprints from non-drug users.

In order to explore the relationship between fingerprints and oral fluid, patients were also asked to take an oral fluid test using a Quantisal™ collection kit (Alere Toxicology, UK). Analysis of oral fluid samples were carried out by Claritest (Norwich, UK). The samples were screened for multiple drugs including cocaine using immunoassay testing. Positive immunoassay screening were confirmed using LC-MS/MS.

## Supplementary information

Supplementary Information.

Supplementary Dataset 1.

## Data Availability

The datasets generated and analysed during the current study are available from the corresponding author on reasonable request.
