# Supplementary material for: On the relevance of cocaine detection in a fingerprint
Source: Sci Rep. 2020 Feb 6;10:1974. doi: 10.1038/s41598-020-58856-0 (PMC7005170; doi:10.1038/s41598-020-58856-0)
Supplement: Supplementary file 1 — Supplementary Information. [file 41598_2020_58856_MOESM1_ESM.docx]

On the relevance of cocaine detection in a fingerprint

M Jang, C Costa, J Bunch, B Gibson, M Ismail, V Palitsin, R Webb, M. Hudson and M J Bailey

# **Supplemental Data Table 1.** Mass Spectrometry parameters used for the paper spray high resolution mass spectrometry method (Thermo Scientific^TM^ Q-Exactive Plus Orbitrap^TM^).

| **Parameter** | **Operating Conditions** |
| --- | --- |
| **Electrospray source parameters** | |
| **Sheath gas flow rate** | 45 |
| **Aux gas flow rate** | 10 |
| **Spray voltage** | 4 kV |
| **Capillary temperature** | 250 °C |
| **S-Lens RF level** | 50 |
| **Full Scan** | |
| **Scan type** | Full MS - SIM |
| **Scan rage** | *m/z* 66.7 – 1000 |
| **Resolution** | 280,000 at *m/z* 200 |
| **Polarity** | Positive |
| **AGC target** | 10^6^ |
| **Maximum inject time** | 500 ms |
| **MS/MS** | |
| **Scan type** | Parallel Reaction Monitoring (PRM) |
| **Resolution** | 17,500 |
| **AGC** | 200,000 |
| **Max inject time** | 50 ms |
| **Isolation window** | 1.0 m/z |
| **NCE** | 45 |

**
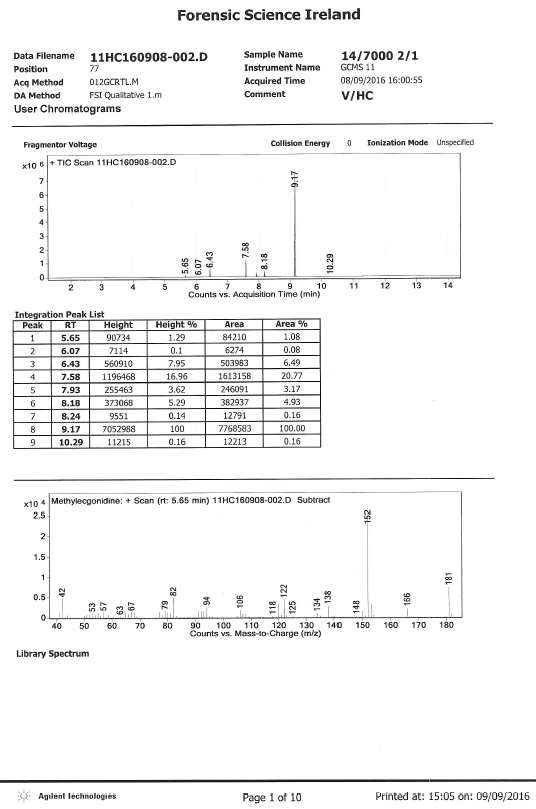
**

- Cocaine

- Benzoylecgonine

- Lidocaine

- Caffeine

- Benzocaine

- Ecgonine Methyl Ester

- Methylecgonidine

**A**


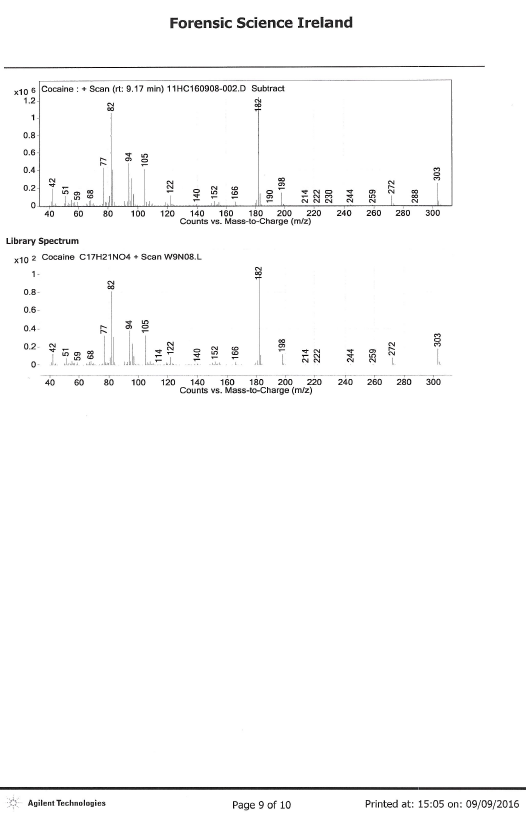


Cocaine

Cocaine fragments

**B**


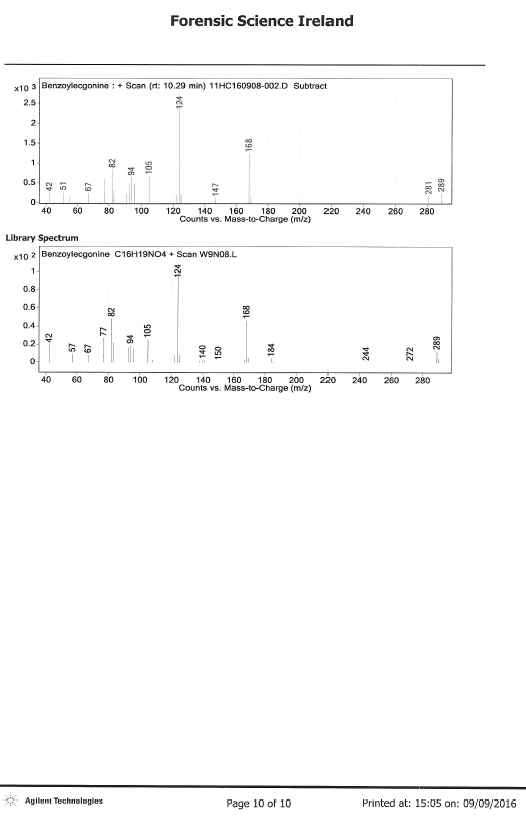


Benzoylecgonine

Benzoylecgonine fragments

**C**

# **Supplement Data Figure 1.** Gas chromatography-mass spectrometry (GC-MS) results of the analysis of seized cocaine samples by Forensic Science Ireland (FSI). (A) Total ion chromatogram (TIC) of the sample analysed; (B) MS/MS spectrum of cocaine peak, detected at 9.17 min; (C) MS/MS spectrum of benzoylecgonine peak, detected at 10.29 min.


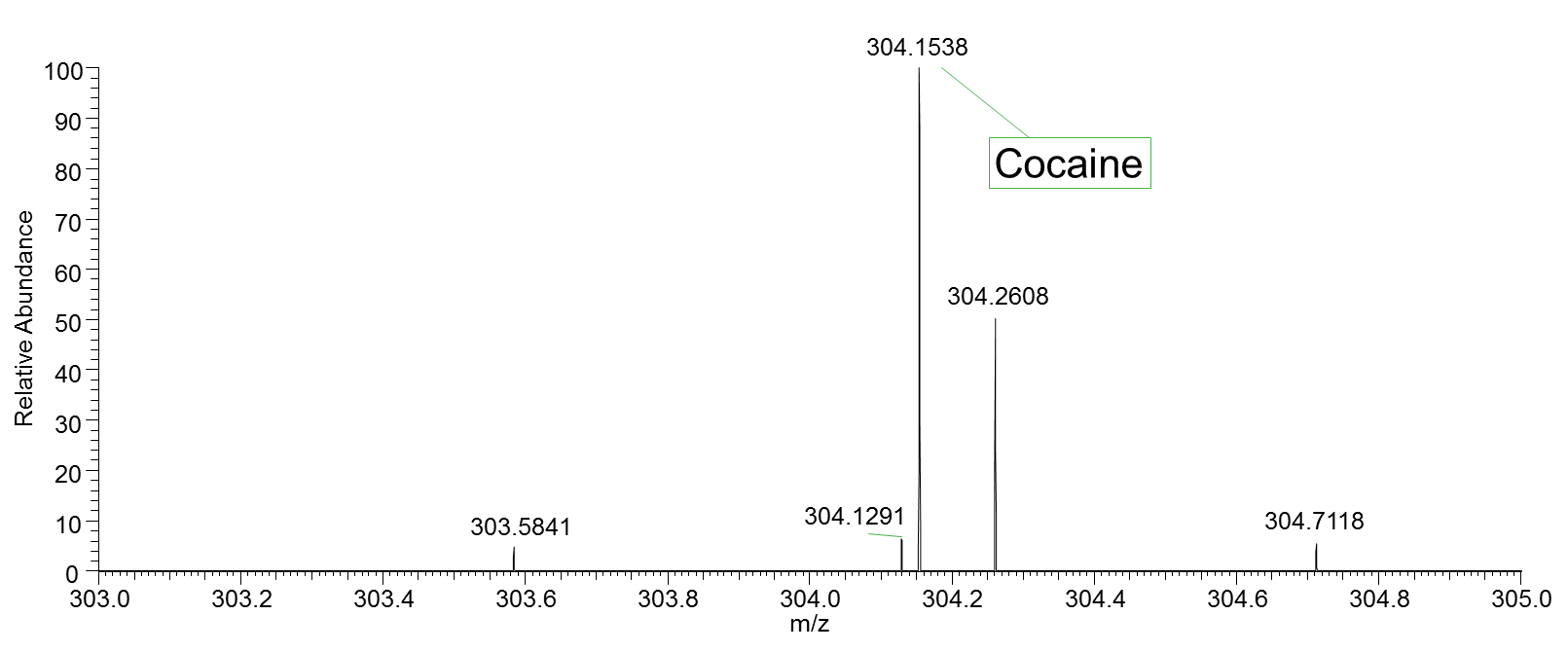


**A**


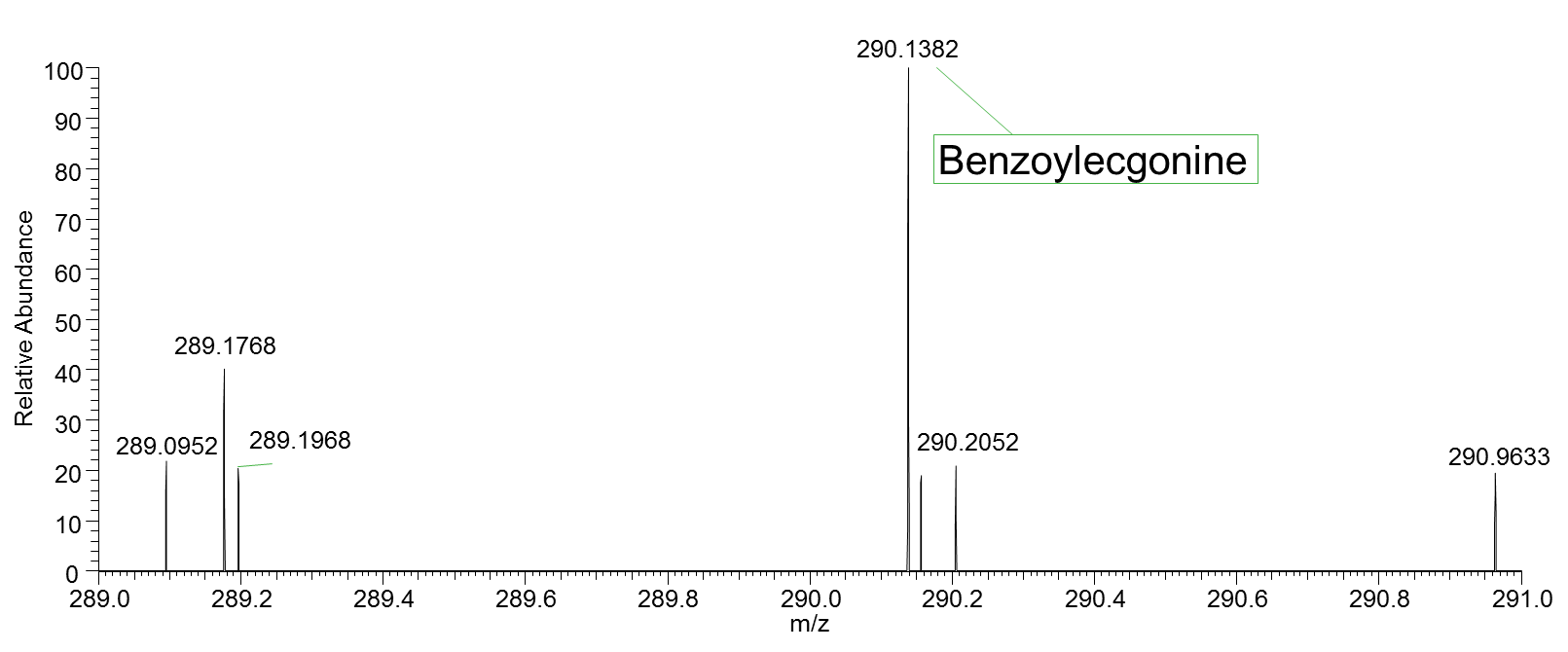


**B**


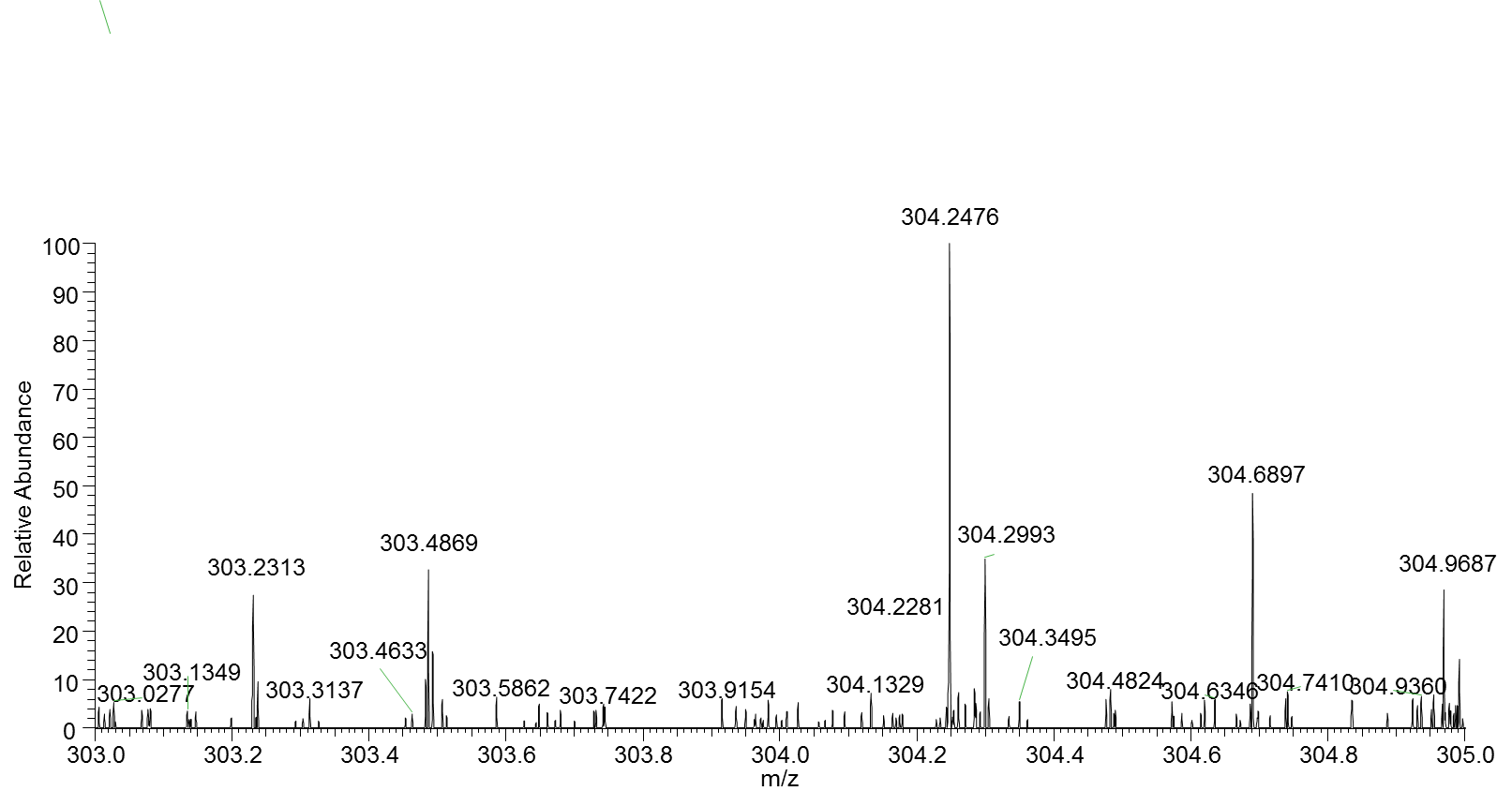


**C**


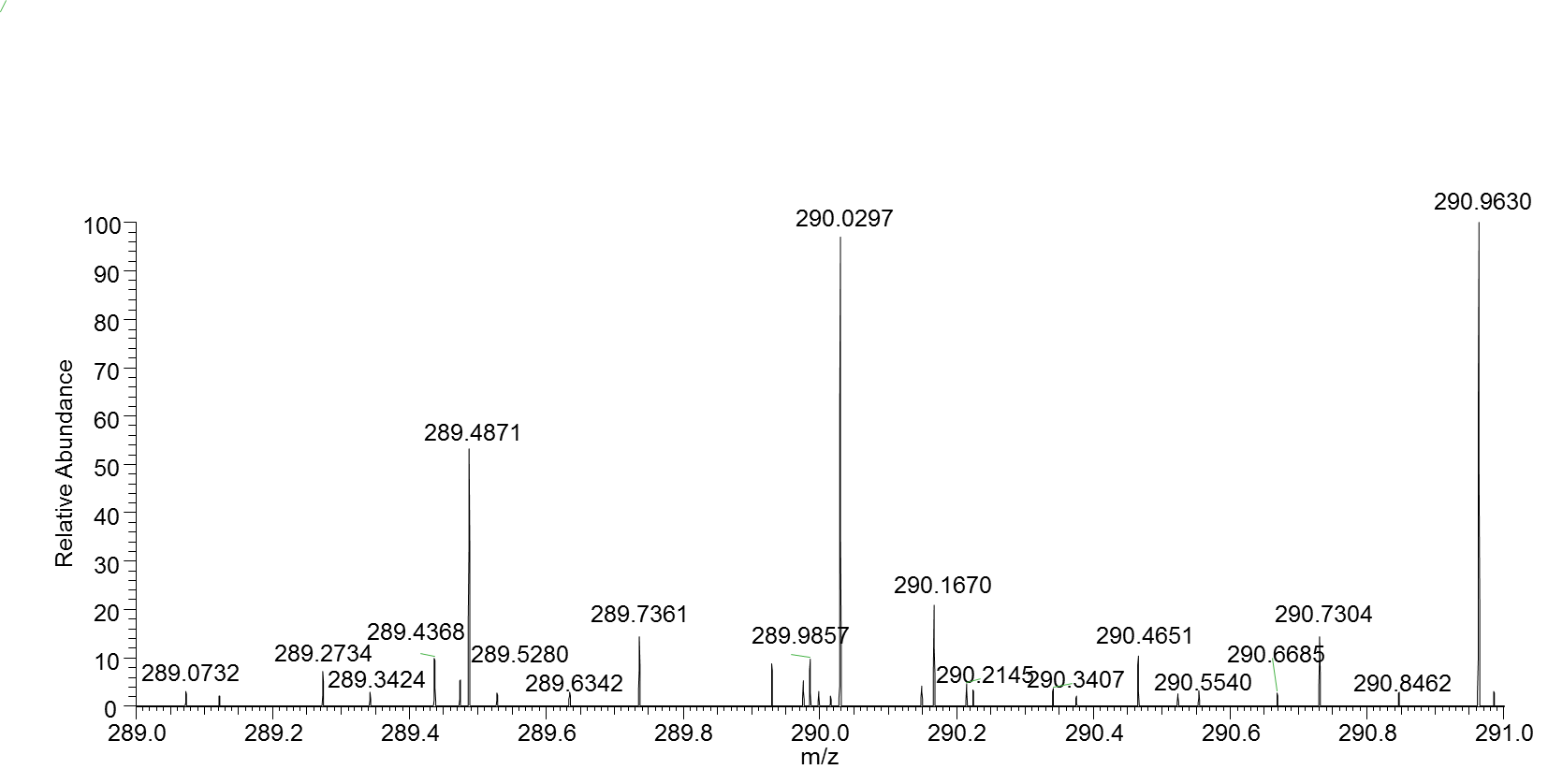


**D**


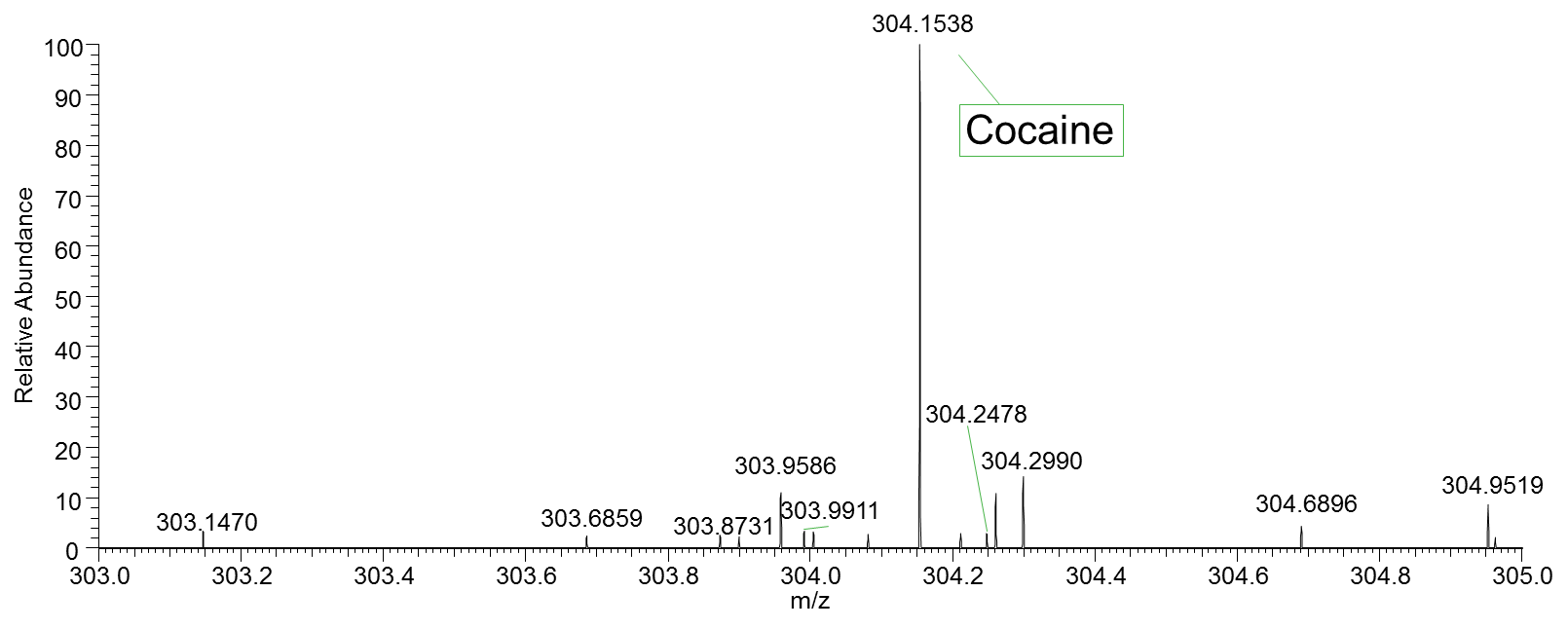


**E**


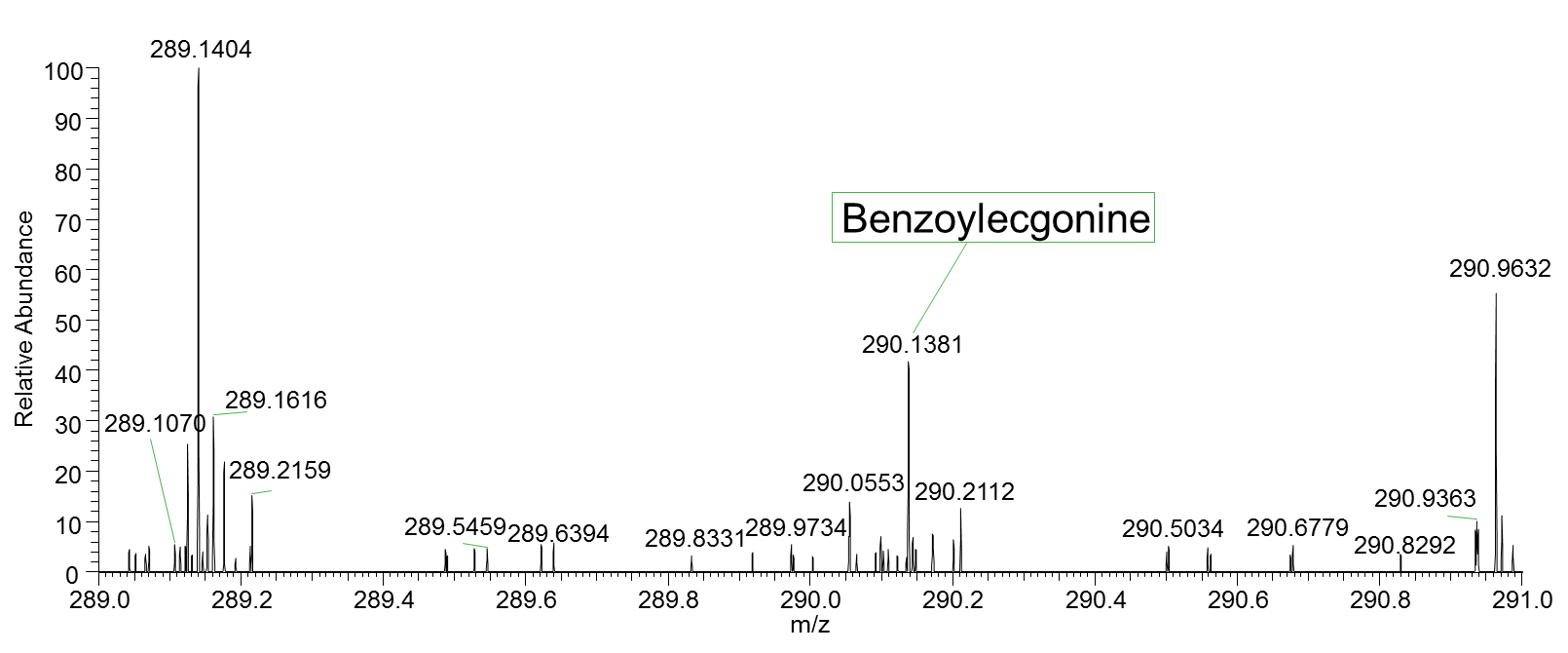


**F**

# **Supplemental Data Figure 2.** Example mass spectra for cocaine (*m/z* 304.1539) and Benzoylecgonine (BZE, *m/z* 290.1380) from a standard (300 pg) of cocaine and BZE (A&B), a fingerprint from a non-drug user (C&D), and a fingerprint from a drug user (E&F) generated using the paper spray high resolution mass spectrometry method outlined in Figure 1, main paper. Peak assignment was confirmed by agreement with standard to 1 ppm and fragment ion peaks using MS/MS (see Supplementary Figure 2).


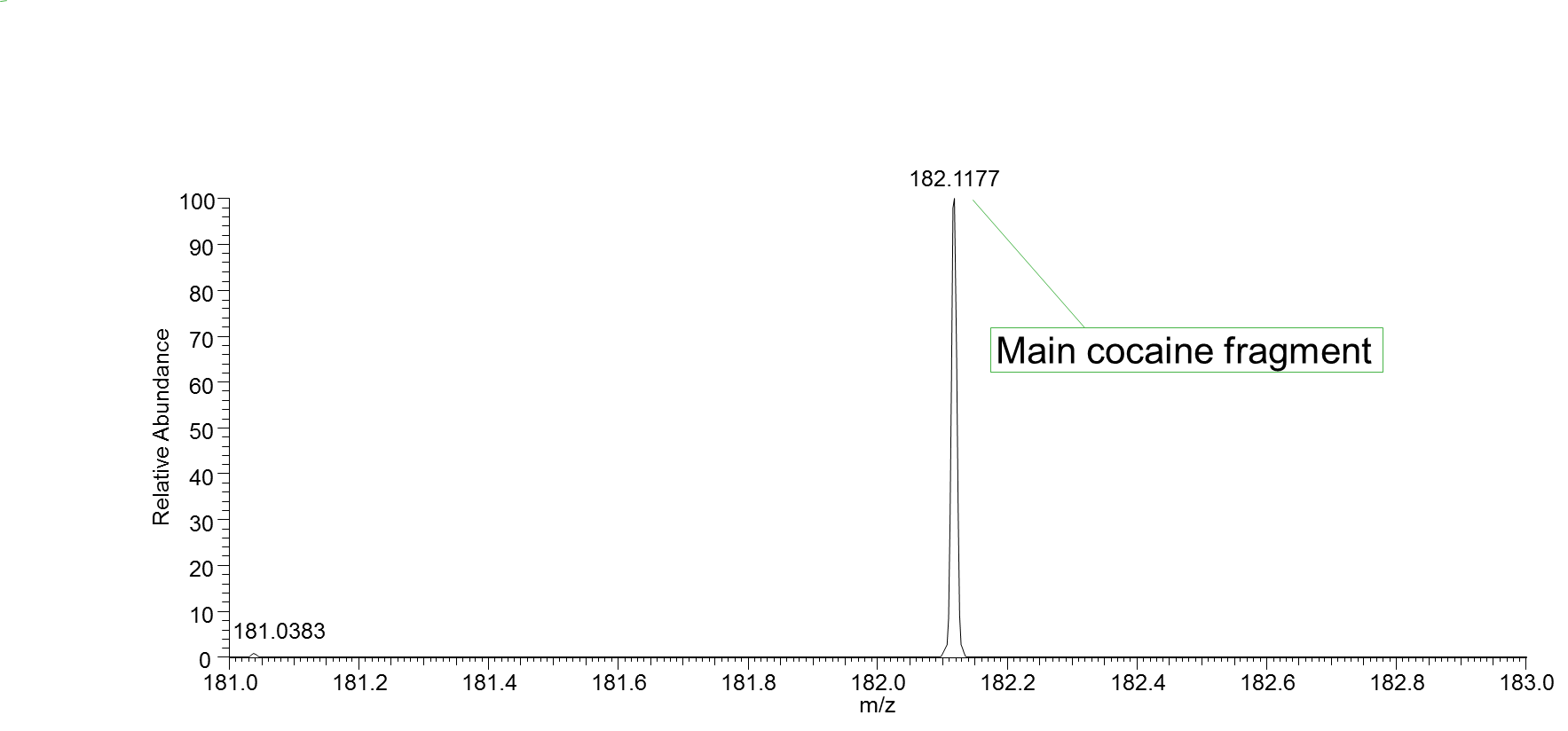


**A**


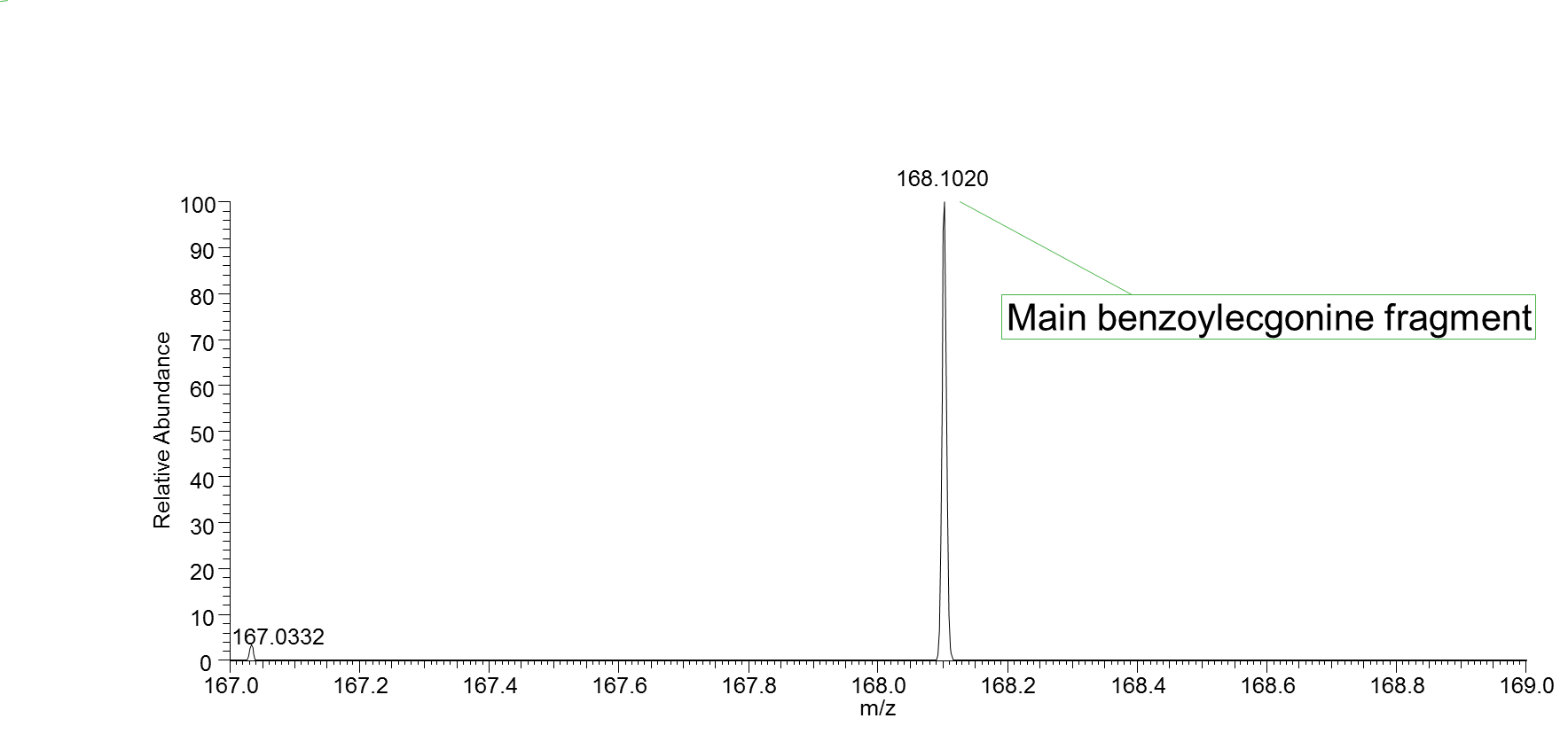


**B**


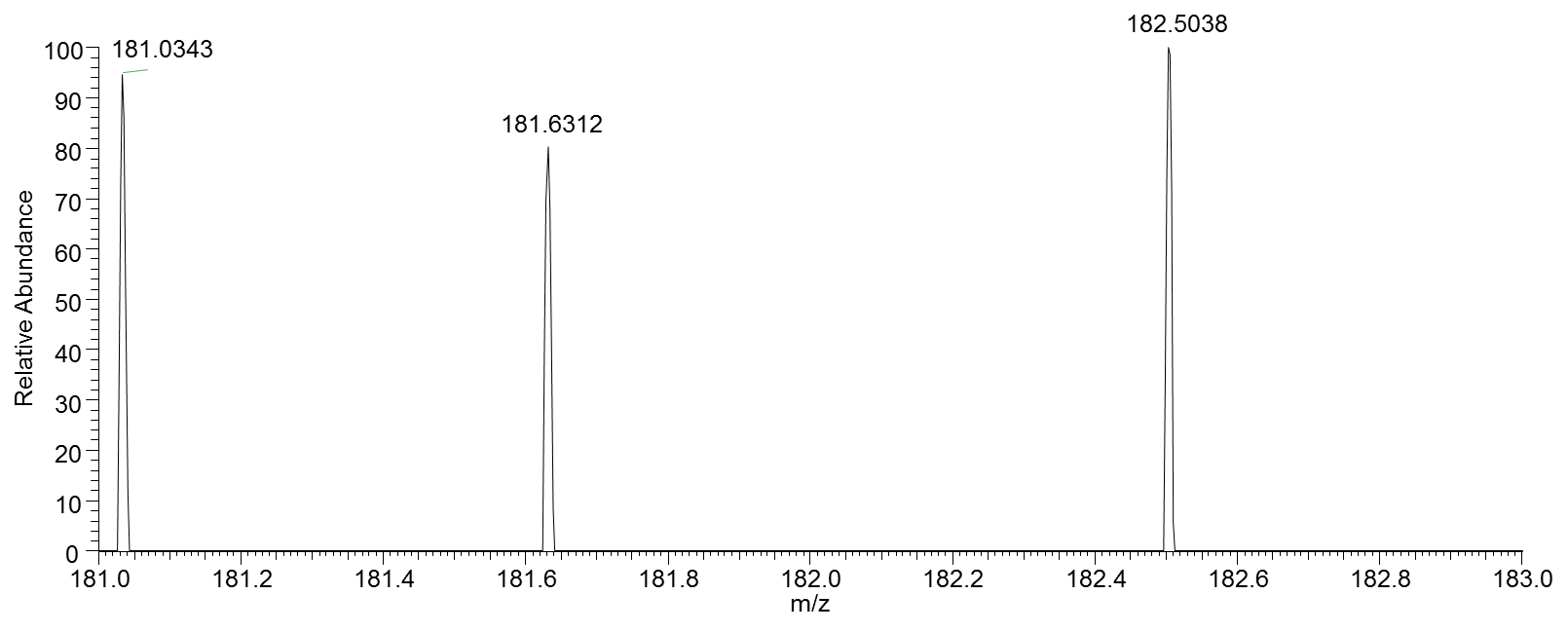


**C**


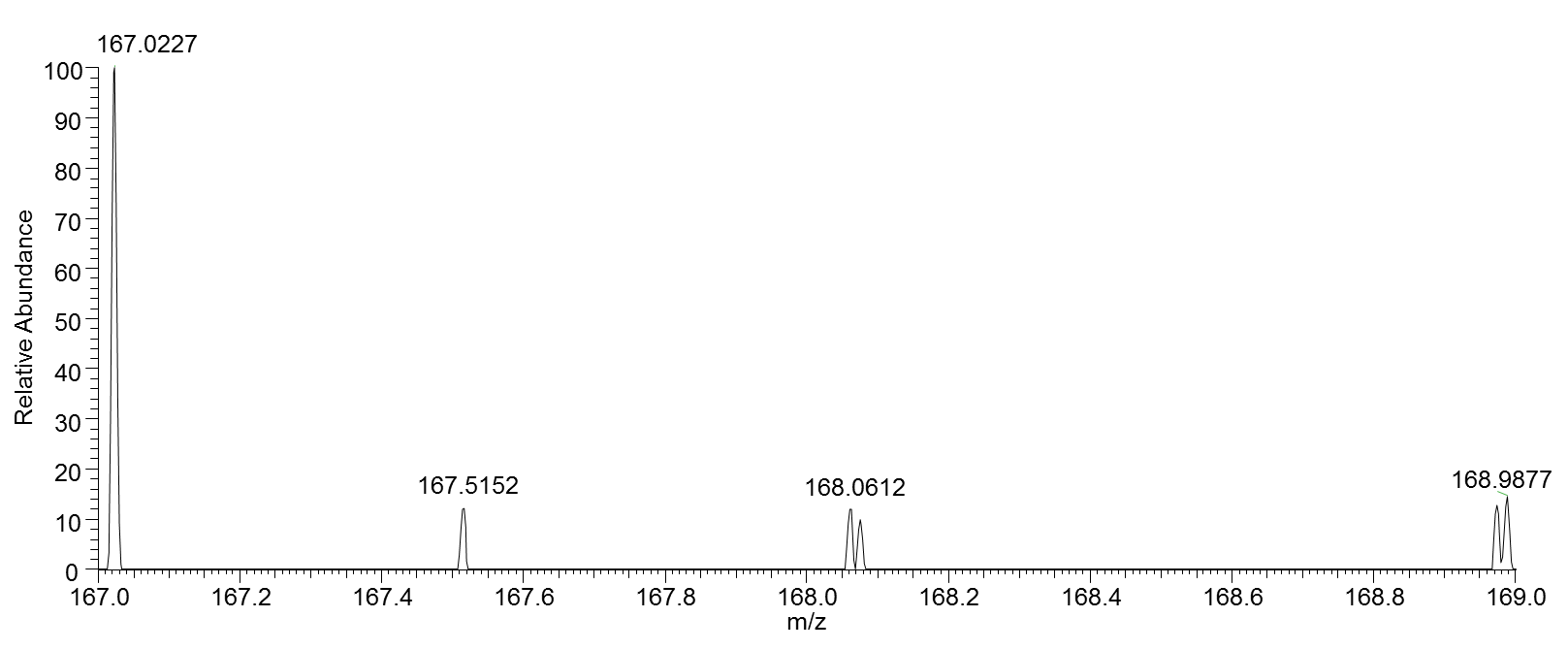


**D**


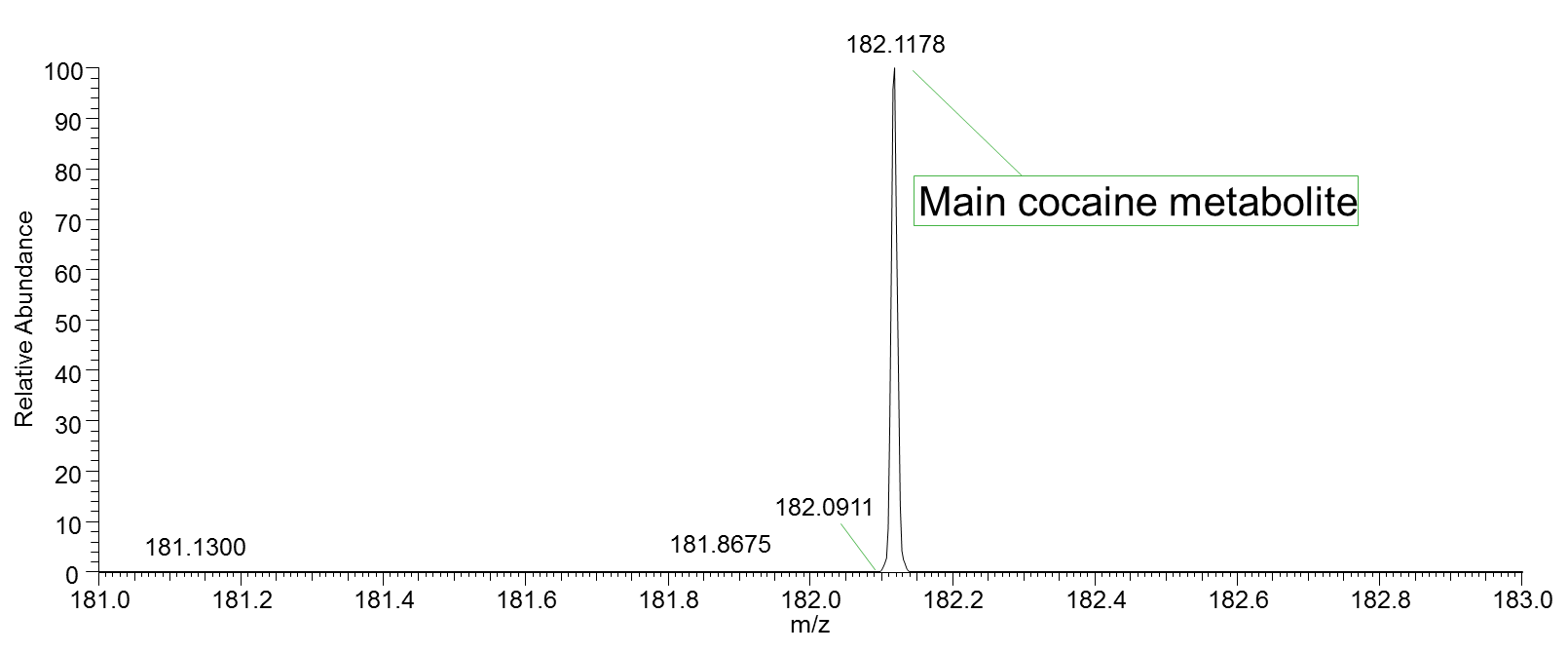


**E**


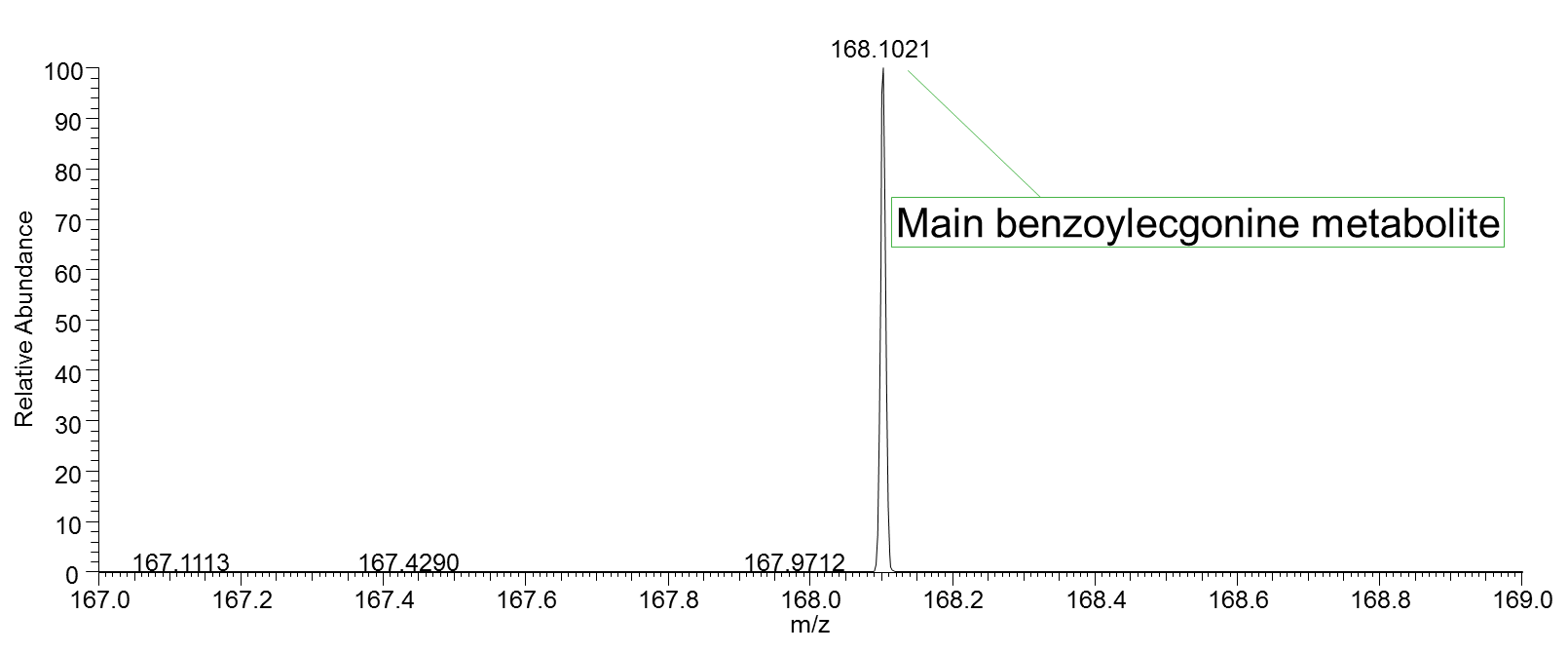


**F**

# **Supplemental Data Figure 3.** Example MS/MS spectra for cocaine (*m/z* 304.1>182.1) and Benzoylecgonine (BZE, *m/z* 290.1>168.1) from a standard (300 pg) of cocaine and BZE (A&B), a fingerprint from a non-drug user (C&D), and a fingerprint from a drug user (E&F) generated using the paper spray high resolution mass spectrometry method outlined in Figure 1, main paper. Peak assignment was confirmed by agreement with standard to within 5 ppm.

# **
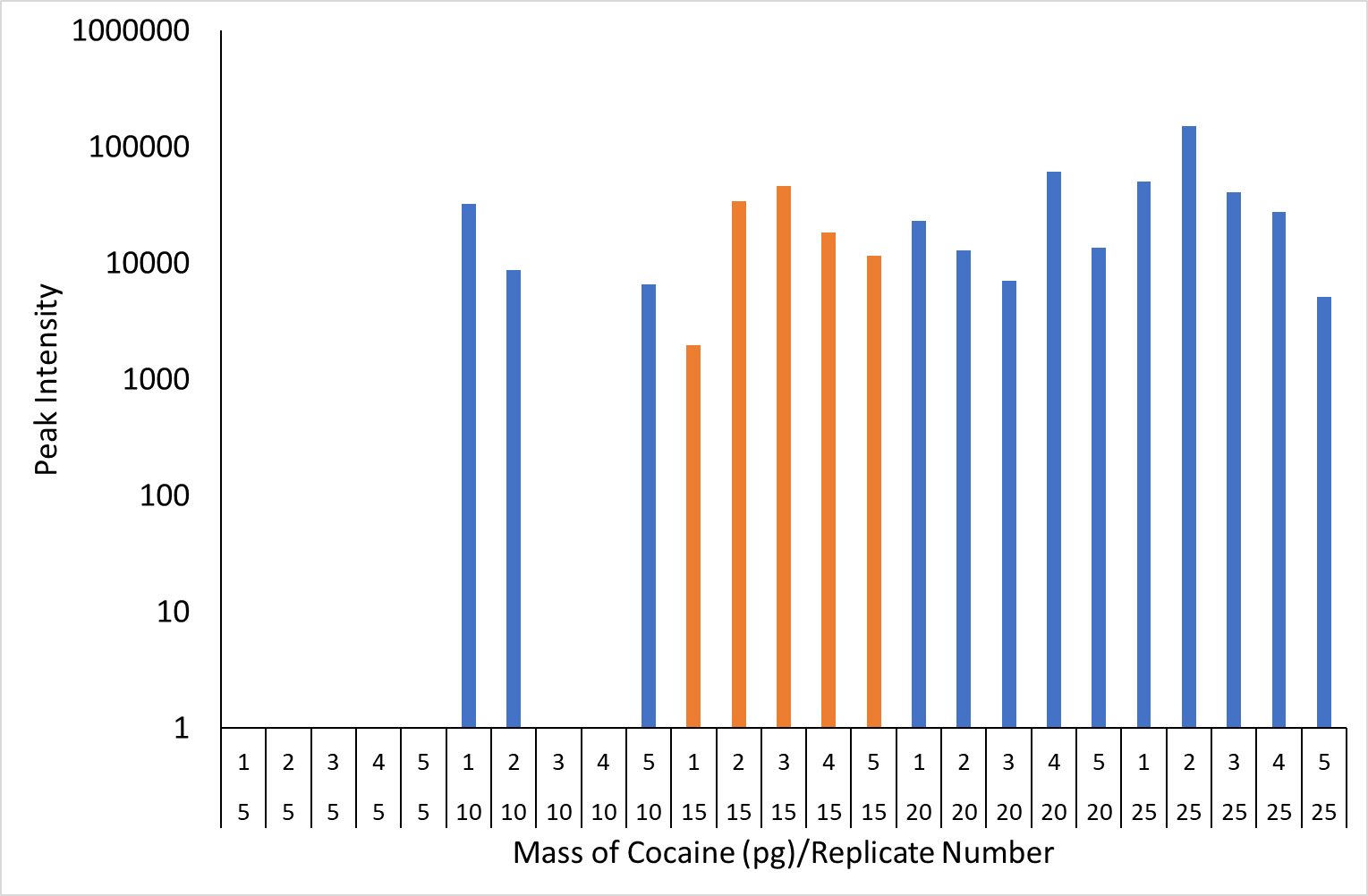
**

**A**

# **Supplemental Data Figure 4.** Results from infinite dilution experiment to determine the limit of detection for cocaine (A). The data is plotted as the peak intensity of the protonated molecular ion of analyte. Based on 5 replicate measurements using the paper spray mass spectrometry method outlined in Figure 1, main paper.

**A**

**A**

**B**

# **Supplemental Data Figure 5.** Calibration curves corresponding to cocaine and BZE between 0 and 400 pg, generated using high resolution paper spray mass spectrometry, demonstrating linearity. The LOQ was determined as the concentration below which the precision was >20%. Based on 3 replicate measurements.

# **Supplemental Data Table 2.** Limits of detection (LOD) and Limits of quantification (LOQ) obtained for the paper spray high resolution mass spectrometry method.

|  | Cocaine | BZE |
| --- | --- | --- |
| **LOD (pg)** | 15 | 50 |
| **LOQ (pg)** | 50 | 100 |

**A**

**B**

# **Supplemental Data Figure 6.** Calibration curves corresponding to cocaine and BZE between 0 and 1200 pg, generated using high resolution paper spray mass spectrometry, showing linearity in this increased range.

# **Supplemental Data Table 3.** Intra and Inter-day precision obtained for cocaine and BZE deposited at QC levels of 100 (cocaine), 600 and 1,200 pg (cocaine and BZE) using the paper spray high resolution mass spectrometry method. Based on 5 replicate measurements.

| **Analyte** | **QC mass (pg)** | RSD (%), Accuracy (%) | | | |
| --- | --- | --- | --- | --- | --- |
|  |  | Day 1 (n=5) | Day 2 (n=5) | Day 3 (n=5) | Inter-day (n=3) |
| **Cocaine** | **100 pg** | 20%  86% | 15%  100% | 17%  88% | 4%  97% |
|  | **600 pg** | 9%  89% | 10%  107% | 11%  106% | 6%  99% |
|  | **1200 pg** | 11%  86% | 5%  125% | 6%  85% | 6%  101% |
| **BZE** | **600 pg** | 16%  75% | 9%  86% | 20%  77% | 5%  76% |
|  | **1200 pg** | 14%  82% | 12%  87% | 19%  94% | 22%  92% |

**B**

**A**

# **Supplemental Data Figure 7.** Intensity ratio analyte to internal standard (A/IS) of protonated molecular ions corresponding to (A) cocaine and (B) BZE in the presence of standard only (std only), compared with a single fingerprint from each of three donors, used to evaluate matrix effects. Based on n=5 replicates.

# **Supplement Data Figure 8.** Analyte-to-internal standard ratio (A/IS) of protonated molecular ions corresponding low and high QC standard (100 and 1200 pg) cocaine and BZE aged under two different conditions (5 °C and room temperature) analysed immediately after deposition on paper (T=0 days) and after 3 weeks (T=21 days) to evaluate stability. Based on n=3 replicates. Application of a T-test (p=0.05) showed no significant difference found between T=0 and T=21 days.
